# Integrating Host Genetics and Clinical Setting in Machine Learning Models: Predicting COVID-19 Prognosis for Healthcare Decision-Making (The FeMiNa Study)

**DOI:** 10.3390/diagnostics16040583

**Published:** 2026-02-15

**Authors:** Elisabetta D’Aversa, Bianca Antonica, Miriana Grisafi, Rosanna Asselta, Elvezia Maria Paraboschi, Angelina Passaro, Stefano Volpato, Francesca Remelli, Massimiliano Castellazzi, Alberto Maria Marra, Antonio Cittadini, Roberta D’Assante, Francesca Salvatori, Ajay Vikram Singh, Salvatore Pernagallo, Veronica Tisato, Donato Gemmati

**Affiliations:** 1 Department of Translational Medicine, University of Ferrara, 44121 Ferrara, Italy; elisabetta.daversa@unife.it (E.D.); bianca.antonica@unife.it (B.A.); miriana.grisafi@unife.it (M.G.); francesca.salvatori@unife.it (F.S.); 2 Department of Biomedical Sciences, Humanitas University, 20072 Pieve Emanuele, Italy; 3 Medical Genetics and RNA Biology Unit, IRCCS Humanitas Research Hospital, 20089 Rozzano, Italy; 4 Department of Medical Science, University of Ferrara, 44121 Ferrara, Italy; 5 Geriatrics Unit, Sant’Anna University Hospital of Ferrara, 44124 Cona, Italy; 6 Department of Neurosciences and Rehabilitation, University of Ferrara, 44121 Ferrara, Italy; massimiliano.castellazzi@unife.it; 7 University Strategic Centre for Studies on Gender Medicine, University of Ferrara, 44121 Ferrara, Italy; 8 Department of Translational Medical Sciences, Federico II University, 80131 Naples, Italy; 9 Interdepartmental Centre for Research in Gender Medicine-GENESIS, Federico II University, 80131 Naples, Italy; 10 Department of Chemical and Product Safety, German Federal Institute for Risk Assessment (BfR), 10589 Berlin, Germany; 11 DESTINA Genomica S.L, PTS Granada, Edificio BIC, 18016 Armilla, Spain; 12 Laboratory for Advanced Therapy Technologies (LTTA), University of Ferrara, 44121 Ferrara, Italy; 13 Centre Haemostasis and Thrombosis, University of Ferrara, 44121 Ferrara, Italy

**Keywords:** machine learning, artificial intelligence, COVID-19, predictive model, host genetics, healthcare solutions

## Abstract

**Highlights:**

**What are the main findings?**

- eXtreme Gradient Boosting f1 optimization avoids losing several patients due to misdiagnosis.
- Genetic data implement the model’s power for mortality prediction.

**What are the implications of the main findings?**

- Integrating genetics in ML enables a more personalized medical approach.

**Abstract:**

**Background/Objectives**: COVID-19 has made a tremendous impact, causing a massive number of deaths worldwide. The inadequacy of health facilities resulted in shortage of resources and exhaustion of frontline workers who had to manage in a short time many patients with no tools to prioritize those at high risk. This study intended to disclose the architecture of such complex disease and enhance the management of hospitalized patients, preventing severe outcomes. **Methods**: We performed a retrospective multicenter study aimed at refining the best predictive model for COVID-19 mortality, integrating 19 genetic and 13 clinical features. We trained three machine learning (ML) models (GBM, XGB and RF) on a dataset of 532 COVID-19 hospitalized Italian patients, among the 605 recruited during the first wave of the pandemic, when vaccines were not available. **Results:** All the models achieved great values for accuracy, AUROC, f1, f2 and PR-AUC metrics. XGB f1 optimization resulted in better performance providing fewer false positives (N_f1_ = 26 versus N_f2_ = 27, N_PR-AUC_ = 29), and mostly false negatives (N_f1_ = 63 versus N_f2_ = 69, N_PR-AUC_ = 69), being the main goal to answer. We next delved into the feature importance to understand which features contribute to the model decision: age was the main driver of mortality prediction, followed by ventilation. The remainder was equally distributed between genetic (*HLA-DRA* rs3135363, *PPARGC1A* rs192678, *CRP* rs2808635, *ABO* rs657152) and other clinical features, demonstrating that genetic data did not confound, but rather implemented, the power of the model. **Conclusions**: Our results suggest that integrating genetic and clinical data into ML models is crucial for identifying high-risk cases within the vast disease heterogeneity, enabling the P4-medicine approach to improve patient outcomes and support the healthcare system.

## 1. Introduction

SARS-CoV-2 is the seventh encountered virus strain causing respiratory disease in humans, ranging from mild to severe symptoms [1,2]. Age, male sex, and defined comorbidities, including cardiovascular and pulmonary disease, appear to increase the severity and mortality of COVID-19 [3,4,5,6]. Nevertheless, low-risk individuals, such as young people without comorbidities, may evolve with severe disease, needing hospitalization [7]. Overall, about 5% of COVID-19 patients were admitted to the intensive care unit (ICU), 2.3% underwent invasive mechanical ventilation and 1.4% died [8]. From the beginning of the pandemic, considerable efforts have been made to identify informative indicators and risk factors influencing SARS-CoV-2 susceptibility and COVID-19 prognosis [4,9]. The extreme clinical heterogeneity observed in patients and the different geographical distribution of cases cannot be explained solely by the presence or absence of possible concomitant health conditions. This observation strongly suggests that individual host genetics may play a crucial role in determining clinical phenotypes across populations [5,10,11]. Several Genome-Wide Association Studies (GWAS) and international initiatives have identified hundreds of gene variants, most of them Single Nucleotide Polymorphisms (SNPs) and haplotypes that are causative of the severe phenotype or confer an advantage in fighting the infection [10,12,13,14,15]. In this context, several genes involved in inflammatory response (Interleukin6 (*IL6*), Tumor Protein p53 (*TP53*), C-Reactive Protein (*CRP*), C-C motif Chemokine Receptor 2 (*CCR2*)), immune response (Human Leukocyte Antigen (*HLA*), Complement factor H (*CFH*)), susceptibility to viral infections (3′,5′-Oligoadenylate Synthase 3 (*OAS3*), (Leucine zipper transcription factor-like 1 (*LZTFL1*), Cystic Fibrosis Transmembrane Conductance Regulator (*CFTR*)), or genes implicated in energy metabolism (Apolipoprotein E (*APOE*), PPARG coactivator 1 alpha (*PPARGC1A*), Uncoupling Protein 1 (*UCP1*)) and in the entrance of SARS-CoV-2 (Angiotensin converting enzyme 2 (*ACE2*)) have been investigated (see Table 1 for the specific References).

COVID-19 has made a tremendous impact on the health of people all over the world and caused a significant number of deaths, heavily impacting health systems [16,17]. In Italy 27 million cases and 198,000 deaths were documented, with daily hospitalization costs ranging from €475.86 to €1401.65 for the most severe ICU patients [18,19]. All past experiences should help to better understand the critical areas for improvement and set up systems and mandatory prevention measures to counteract future infections and manage other burden diseases. What emerged most was undoubtedly the inadequacy of health facilities, resulting in a severe shortage of medical resources and the exhaustion of frontline healthcare workers [20,21] who had to manage many patients in a short time without any tools to calculate specific diagnoses and prognoses in advance. The unpredictable disease course made diagnosis and treatment a challenging mission, particularly for high-risk patients. Hence, the early identification of those at risk is necessary to mitigate the burden on healthcare delivery and reduce mortality as much as possible [22,23].

Analytical models that accurately predict COVID-19 outcomes could assist in efficiently allocating the limited medical resources, improving the healthcare quality, and eventually optimizing patient management [24]. Predictions from various computational and statistical models have been used to address this challenge [25,26]. Since the beginning of the COVID-19 pandemic, new digital technologies, such as artificial intelligence (AI) and machine learning (ML), have been introduced as predictors of COVID-19 mortality [27]. In recent years, their intersection with healthcare has revolutionized disease prediction, delivering more accurate results than conventional statistical models particularly for complex diseases, deciphering relationships between biomarkers and symptoms, and predicting clinical outcomes. Generally, the prognostic performance of ML-based models for predicting COVID-19 outcomes (e.g., mortality, length of hospitalization, ICU admission, or long COVID) [28,29,30] utilizes conventional risk factors such as comorbidities, clinical manifestations, laboratory results, and demographic characteristics [31,32,33].

In 2020, the COVID-19 Host Genetics Initiative (HGI) brought together the human genetics community to generate, share, and analyze epidemiological and scientific data to understand the genetic bases and determinants of COVID-19 susceptibility, clinical severity, and prognosis (https://www.covid19hg.org/, accessed on 10 December 2025). Our research group took part in the HGI with the project “Extreme genotype-phenotype comparison in SARS-CoV2 patients: direct candidate genes Pathway and GWAS”, aimed at identifying candidate genes and variants related to pathways specifically involved in COVID-19 [3,34,35]. Subsequently, our group also moved toward facing this complex painting by a COVID-omics strategy and AI to gain future perspectives in tailored diagnosis, therapeutic schemes, vaccine efficacy, and other associated coinfections [36,37,38,39,40].

With growing global knowledge about COVID-19 and considering that both genetic and clinical features can contribute to its complexity, our attention has unavoidably shifted toward developing an informative predictive model of the disease course. We considered the effects of the main genetic and acquired factors and how their combined mutual interactions could change susceptibility and predispose to severity. In the present retrospective multicenter investigation, we optimized and compared different ML approaches to develop accurate algorithms for mortality risk and long hospital stay prediction in COVID-19 patients. They were recruited from three different Italian centers (Ferrara, Fe; Milan, Mi; Naples, Na; the FeMiNa study) during the first wave of the pandemic and before any efficient treatment, or drug, or vaccine was available. Subsequently, we analyzed the interactions among the variables, focusing on the contribution of the genetic component. This approach could also help us to unravel the complex architecture of such infectious diseases and extract meaningful patterns and insights where the host genetics, in addition to clinical factors, can influence susceptibility and prognosis.

## 2. Materials and Methods

### 2.1. Study Participants and Sample Recruitment

We recruited 605 Italian patients among those hospitalized after confirmed SARS-CoV-2 infection who developed severe COVID-19 symptoms during the period January–September 2020, far from vaccine availability. During the study period, the cumulative incidence of COVID-19 cases was 3740.15 per 100,000 inhabitants, 4673.71 per 100,000, and 3175.57/100,000 in the regions where Ferrara, Milan and Naples are located (Emilia Romagna, Lombardia and Campania respectively), with 5% hospitalization rate (updated to 31 December 2020 [41]). After signed informed consent and local ethical committee approval (IRB) were obtained, whole blood samples were collected (N = 300 from the Hospital-University of Ferrara, CE:405/2020; N = 182 from the IRCCS Humanitas Clinical and Research Center of Milan, CE:316/2020; N = 123 from the Federico II University Hospital of Naples, CE:98/2021). At patient admission, personal anamnesis including existing comorbidities and other characteristics have been recorded (i.e., hypertension, heart failure, ischemic stroke, arteriopathy, Chronic Obstructive Pulmonary Disease (COPD), hepatopathy, neoplasm, dementia, diabetes, sex, age, need for ventilation and blood type).

### 2.2. Genomic DNA Extraction

Whole blood samples have been collected in vacutainer containing EDTA as anticoagulant. All the samples were anonymized using alpha-numeric encryption, with the decryption keys available only to the PIs and co-PIs of the respective centers where patients were recruited. Blood samples were handled in Bio-Safety Level P3 cleanrooms and stored at −40 °C until genomic DNA extraction. DNA was isolated using an automated DNA extraction and purification robot (BioRobot EZ1 system, Qiagen; Hilden, Germany). For those samples with low DNA extraction yield, a different extraction procedure was employed using the QIAamp DNA Blood Mini Kit columns (Qiagen).

### 2.3. Genotyping and Variant Analysis

Detection of the selected gene variants was performed by qPCR using rhAmp SNP genotyping technology (IDT, Integrated DNA Technologies; Coralville, IA, USA) for the following SNPs: *ACE2* rs2285666, *HLA-A* rs2499, *TP53* rs1042522, *OAS3* rs10735079, *LZTFL1* rs35044562, *IL6* rs2228145; by TaqMan SNP genotyping Assay (Thermo Fisher Scientific, Applied Biosystems; Waltham, MA, USA) for the following: *APOE* rs429358, *APOE* rs7412, *PPARGC1A* rs8192678, *HLA-DPB1* rs9277356, *HLA-DRA* rs3135363, *UCP1* rs1800592, *IL6* rs1800795, *CRP* rs2808635, *CRP* rs876538, *CFH* rs1061170, *CFTR* (rs113993960, rs1801178, rs113993959, rs76713772, rs74597325, rs80034486, rs74767530, rs77010898, rs121908799), on QuantStudio3 Real-Time PCR System (Thermo Fisher Scientific), according to the supplier instruction. Finally, alpha 1-3-N-acetylgalactosaminyltransferase and alpha 1-3-galactosyltransferase (*ABO*) rs657152 and *CCR2* rs34041956 were detected by pyrosequencing (PyroMark Q96 Autoprep ID System, Biotage, AB; Uppsala, Sweden) after standard PCR amplification [42] on Agilent SureCycler 8800 (Agilent Technologies; Santa Clara, CA, USA).

### 2.4. Statistical Analysis

The statistical analysis was performed by the R statistical package (R Core Team, v4.1.2; Vienna, Austria [43]) and Statistical Package for the Social Sciences (SPSS, IBM SPSS Statistics for Windows, v29.0.2.0; Armonk, NY, USA [44]). In the study population, the allele frequencies for each variant were compared with those reported in the National Center for Biotechnology Information (NCBI) registry for the European population. The genotype distributions were further verified for the Hardy–Weinberg Equilibrium. The normal distribution of the variables was evaluated through the Kolmogorov–Smirnov test. Comparisons of categorical variables have been performed using the *t*-test and Χ^2^-test. Pearson correlation was calculated by pairing all the variables with the output and reported as a correlation matrix. The association of a genotype (or allele) with the clinical phenotype of interest has been performed through associations for multiple inheritance models (dominant and recessive). For each model, we calculated Odds Ratio (OR) and the 95% confidence interval (CI) using MedCalc Statistical Software (MedCalc Software, v19.2.6 bv; Ostend, Belgium [45]). *p*-value ≤ 0.05 was considered statistically significant, and this threshold has been set for all the computed analyses.

### 2.5. Principal Component Logistic Regression (PCLR)

Before training ML models, the dataset was analyzed through PCLR as a first predictive statistical approach to determine the combination of clinical and genetic variables in forecasting bad outcomes (i.e., death and long hospital stay (≥15 days) among survivors). Specifically, we combined Principal Component Analysis (PCA) with binary logistic regression using PCs as covariates. Age was scaled before PCA according to the formula [Z = (x − μ)/σ]. Gene variants were coded 1, 2, 3, for common homozygous, heterozygous, and rare homozygous variant respectively. *CFTR* variants were grouped and binary categorized into non-carriers (1) and heterozygous carriers (2). No *CFTR* homozygotes have been found. All clinical features have been categorized as binary codes (0/1), except for blood type and ventilation (see Table 2). The PCs with eigenvalue exceeding 1.0 have been computed in a binary logistic regression analysis as independent variables. The relationship with the dependent variable was considered statistically significant with *p*-value ≤ 0.05. Variables with a loading above the cut-off point ±0.3 were considered to be dominant in a component.

### 2.6. Machine Learning Assessment

We employed three ML algorithms, Random Forest (RF) [46,47], Gradient Boosting Machine (GBM) [48], and eXtreme Gradient Boosting (XGB) [49], to predict mortality and hospital stay among the cohort of COVID-19 patients, and the outputs were further compared. Specifically, the ML analyses were conducted in cooperation with an external consultants (Service DIMEC, Bologna, Italy).The implementation of these algorithms was provided by the H2O.ai framework (Python Interface for H2O, H2O.ai, v3.46.0.3, July 2024; Mountain View, CA, USA [50]). Each algorithm was designed to enhance the different aspects of predictive accuracy and computational efficiency. In detail, RF builds multiple decision trees and merges them to obtain a more accurate and stable prediction; GBM builds a collection of decision trees in a sequential manner, where each subsequent tree corrects the errors of the previous ones; XGB is an implementation of GBM that, by providing parallel tree boosting, is known for its efficiency and performance.

Globally, our study analyzed a dataset consisting of 34 features, divided into 19 categorical gene variants, 13 clinical/epidemiological variables and two different outcomes. Among the clinical/epidemiological features, 12 were categorical (pure nominal, i.e., sex, blood type, and comorbidities; categorical ordinal, i.e., ventilation), while age was the only one computed as a continuous variable. The primary outcome (endpoint 1), that is whether or not patients died of SARS-CoV2 infection (i.e., 0:alive or 1:dead), was a categorical variable (binary); the secondary outcome (endpoint 2), the length of hospital stay before discharge or death, was instead continuous. Patients lacking more than 20% data were excluded to ensure data integrity, resulting in a final sample of 532 patients.

Regarding endpoint 1, the dataset exhibited a significant imbalance between the number of deceased cases (N = 92; 17.3%) and survivals (N = 440; 82.7%), imposing a careful selection of evaluation metrics that focused on the minority class. We prioritized metrics such as precision (the proportion of true positive predictions within all positive classifications) and recall (the modelability to identify all actual positives). Additionally, we employed the f1-score and f2-score, which balance precision and recall. The f1-score is the harmonic mean of precision and recall; it integrates these latter into a single metric gaining a better understanding of the model performance and offering a balanced measure between these two. Its optimal value is 1.0, indicative of perfect precision and recall, whereas its minimum value is fixed at 0. Conversely, the f2-score is the weighted harmonic mean of precision and recall. Unlike f1-score, which equally weighs precision and recall, f2-score weighs the latter higher than the former by a factor of 2.0. Area Under the Curve (AUC) and PR curves (PR-AUC) were used to quantify the overall efficacy of our classification model across various thresholds. We also applied data weighting techniques during model training. Data weighting involves assigning different weights to the classes, typically giving more importance to the minority class (deceased cases).

Extensive hyperparameter tuning using GridSearchCV was conducted to systematically explore a wide range of configurations to identify the most effective settings based on our primary evaluation metrics: f1-score, f2-score, and PR-AUC. Specifically, 30 configurations for RF, 432 for the GBM, and 72 for XGB were tested. Specifically, the hyperparameters for the RF model were max_depth, max_features, min_sample _leaf, and n_estimators. For the GBM they were learning_rate, max_depth, max_features, n_estimators, and subsample. In the case of XGB, parameters were booster, colsample_bytree, learning_rate, max_depth, subsample, and n_estimators. In Supplementary Tables S1 and S2, the different hyperparameter values and the results for each ML model have been reported. Given the relatively small size of our dataset, a 10-fold cross-validation approach was implemented for all models, rather than dividing the data into separate training and test sets [51].

Within the H2O.ai framework, the feature importance was calculated based on its attributed reduction in squared error (SE) whenever a node is split using that feature and displayed as SHapley Additive exPlanations (SHAP) plot, showing the features ranked on the y-axis according to their importance, while the x-axis reports the SHAP value. This value indicates the magnitude and direction of each feature contribution to the model prediction.

## 3. Results

### 3.1. Demographic, Clinical and Genetic Characteristics

Those cases lacking more than 20% of variables were not included in the predictive model assessment, which reduced the sample from 605 to 532 individuals, accounting for about 88% of the total recruited. In the analyzed cohort, males (N = 314; 59.0%) were significantly overrepresented (*p*-value = 0.00001) and had a significantly younger mean age than females (67 ± 14 ♂ versus 74 ± 16 ♀; *p*-value < 0.0001) across the global age range of 19 to 99 years. Days of hospitalization vary from 1 day to almost 6 months, with females exhibiting shorter mean values (20.7 ± 20.3 ♂ versus 18.00 ± 16.7 ♀). Alive male patients had longer hospital stays before discharge (21.16 ± 21.13 ♂ versus 18.47 ± 17.46 ♀) despite their significantly younger age (65.42 ± 13.74 ♂ versus 71.18 ± 15.59 ♀; *p*-value < 0.0001). On the other hand, among deceased patients, males and females showed similar lengths of hospital stay before death (17.91 ± 14.94 ♂ versus 15.83 ± 21.75 ♀) despite of a significant younger age in males (76.44 ± 12.77 ♂ versus 84.83 ± 13.48 ♀; *p*-value < 0.0005). This reflects the overall mean age of the two patient cohorts in line with male sex and older age being the combination at greatest risk. Globally, males accounted for a cumulative hospitalization of 6442 days versus females, which lengthened to 3843 days. A combination of gene variants, shown in Table 1, and clinical variables, shown in Table 2, has been computed in the analyses.

**Table 1:** Panel of the selected gene variants investigated.

Gene	SNP (rs)	Nucleotide Change	Reported Frequencies		Observed Frequencies		Ref.
			major allele	minor allele	major allele	minor allele	
*ACE2*	2285666	C>T	0.783	0.216	0.820	0.180	[52,53]
*APOE*	7412	C>T	0.917	0.083	0.933	0.066	[54]
	429358	T>C	0.925	0.074	0.921	0.078	
*TP53*	1042522	C>G	0.736	0.263	0.756	0.243	[55]
*OAS3*	10735079	A>G	0.649	0.351	0.606	0.393	[56]
*LZTFL1*	35044562	A>G	0.926	0.073	0.878	0.121	[57]
*ABO*	657152	C>A	0.624	0.376	0.630	0.369	[58]
*PPARGC1A*	7192678	C>T	0.677	0.323	0.656	0.343	[59]
*CRP*	2808635	T>G	0.717	0.283	0.710	0.289	[38]
	876538	C>T	0.816	0.184	0.808	0.191	[60]
*CFH*	1061170	T>C	0.626	0.374	0.657	0.342	[61]
*HLA-DRA*	3135363	A>G	0.713	0.287	0.642	0.358	[62]
*HLA-DPB1*	9277356	A>G	0.692	0.307	0.689	0.310	[63]
*HLA-A*	2499	G>T	0.863	0.136	0.889	0.110	[60]
*UCP1*	1800592	T>C	0.679	0.320	0.718	0.281	[64]
*IL6*	2228145	A>C	0.612	0.388	0.634	0.365	[65]
	1800795	G>C	0.639	0.369	0.683	0.316	[66,67]
*CCR2*	34041956	G>A	0.940	0.059	0.924	0.076	[68]
*CFTR*	113993960	CTT>-	0.997	0.003	0.991	0.009	[69]
	113993959	G>A	0.9996	0.0004	1.0000	0.0000	
	77010898	G>A	0.9996	0.0004	1.0000	0.0000	
	80034486	C>G	0.9997	0.0003	0.9991	0.0009	
	76713772	G>A	0.9998	0.0002	0.9981	0.0019	
	74597325	C>T	0.9998	0.0002	1.0000	0.0000	
	74767530	C>T	0.9999	0.0001	1.0000	0.0000	
	1801178	A>G	0.9999	0.0001	0.9972	0.0028	
	121908799	AA>G	>0.9999	<0.0001	1.0000	0.0000	

Observed frequencies were calculated for major and minor alleles and the reported ones from the European repository (NCBI).

No statistically significant differences have been obtained for the allele distribution comparing the NCBI-reported and our observed frequencies. All the gene variants considered were under Hardy–Weinberg Equilibrium.

**Table 2:** Panel of the clinical and epidemiological variables investigated.

	Whole Cohort (N = 532)	Males (N = 314)	Females (N = 218)	*p*-Value
Age (mean ± SD)	69.9 ± 15.3	67.1 ± 14.1	73.9 ± 16.1	**<0.0001**
Days of hospitalization (mean ± SD)	19.6 ± 18.9	20.7 ± 20.3	18.0 ± 16.7	0.078
Death (0)	440 (82.7%)	265 (84.4%)	175 (80.3%)	0.22
Death (1)	92 (17.3%)	49 (15.6%)	43 (19.7%)	
Blood type non-O	307 (57.7%)	186 (59.2%)	121 (55.5%)	0.39
*Blood type A*	*223 (41.9%)*	*134 (42.7%)*	*89 (40.8%)*	
*Blood type B*	*52 (9.7%)*	*34 (10.8%)*	*18 (8.3%)*	
*Blood type AB*	*32 (6.0%)*	*18 (5.7%)*	*14 (6.4%)*	
Blood type O	225 (42.3%)	128 (40.7%)	97 (44.5%)	0.68
Hypertension (0)	220 (41.3%)	137 (43.6%)	83 (38%)	0.20
Hypertension (1)	312 (58.6%)	177 (56.4%)	135 (62%)	
Heart failure (0)	483 (90.8%)	289 (92%)	194 (89%)	0.37
Heart failure (1)	47 (8.8%)	25 (8%)	22 (10.1%)	
n.a.	2 (0.4%)	/	2 (0.9%)	
Ischemic stroke (0)	476 (89.4%)	284 (90.5%)	192 (88.1%)	0.56
Ischemic stroke (1)	54 (10.1%)	30 (9.5%)	24 (11%)	
n.a.	2 (0.4%)	/	2 (0.9%)	
Arteriopathy (0)	484 (90.9%)	281 (89.5%)	203 (93.1%)	0.24
Arteriopathy (1)	32 (6.0%)	22 (7%)	10 (4.6%)	
n.a.	16 (3.0%)	11 (3.5%)	5 (2.3%)	
COPD (0)	486 (91.3%)	286 (91.1%)	200 (91.7%)	0.88
COPD (1)	45 (8.4%)	27 (8.6%)	18 (8.3%)	
n.a.	1 (0.2%)	1 (0.3%)	/	
Hepatopathy (0)	509 (95.7%)	302 (96.2%)	207 (95%)	0.38
Hepatopathy (1)	22 (4.1%)	11 (3.5%)	11 (5%)	
n.a.	1 (0.2%)	1 (0.3%)	/	
Neoplasm (0)	450 (84.6%)	270 (86%)	180 (82.6%)	0.26
Neoplasm (1)	77 (14.5%)	41 (13%)	36 (16.5%)	
n.a.	5 (0.9%)	3 (0.95%)	2 (0.9%)	
Dementia (0)	453 (85.1%)	281 (89.5%)	172 (78.9%)	**0.001**
Dementia (1)	74 (13.9%)	31 (9.9%)	43 (19.7%)	
n.a.	5 (0.9%)	2 (0.6%)	3 (1.4%)	
Diabetes (0)	421 (79.1%)	246 (78.3%)	175 (82.3%)	0.49
Diabetes (1)	108 (20.3%)	67 (21.3%)	41 (18.8%)	
n.a.	3 (0.6%)	1 (0.32%)	2 (0.9%)	
Oxygen (nasal cannula)	154 (29.0%)	81 (25.8%)	73 (33.5%)	**0.05** *
Ventilation (NIV + Intubation/ECV)	331 (62.2%)	204 (65.0%)	127 (58.2%)	
*Non-invasive ventilation (NIV)*	*185 (34.8%)*	*113 (36.0%)*	*72 (33.0%)*	
*Intubation/ECV*	*146 (27.4%)*	*91 (29.0%)*	*55 (25.2%)*	
n.a.	47 (8.8%)	29 (9.2%)	18 (8.3%)	

SD, standard deviation; n.a., not available; COPD, Chronic Obstructive Pulmonary Disease; NIV, Non-Invasive Ventilation; ECV, ExtraCorporeal Ventilation. *p*-values < 0.05 are reported in bold. * *p*-values = 0.05 by comparing males versus females only considering NIV and intubation/ECV.

**Figure 1:**
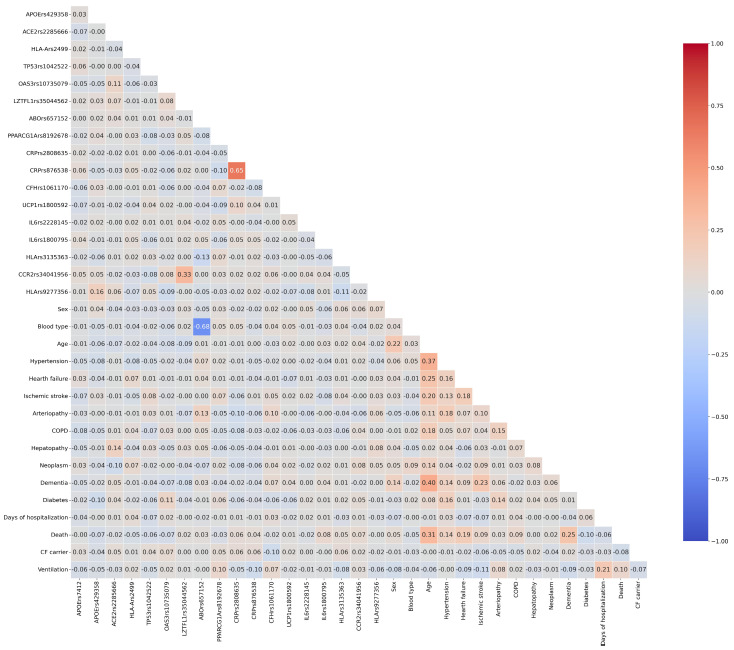
Heatmap of Pearson correlation coefficients among the considered variables. The 34 × 34 diagonal matrix indicates the correlation coefficient (r). The color intensity is proportional to r, with the width of our scale ranging between −0.1 (blue) and 1.00 (red). A cut-off value of 0.5 represents a strong positive correlation. COPD, Chronic Obstructive Pulmonary Disease.

The primary endpoint, death due to COVID-19, has been used as the outcome for further analyses, representing the most severe consequence and progression of the infectious disease, globally reaching an occurrence of 17.3% in our dataset. First, we evaluated all mutual correlations by pairing all variables in a Pearson correlation matrix, as shown in Figure 1.

In the Pearson correlation *CRP* rs2808635 and *CRP* rs876538 reached values > 0.5 because of their verified strong Linkage Disequilibrium (D′ = 0.965) in the European population, as well as *ABO* rs657152 with the phenotypic blood group. Interestingly, any feature shows strong positive or negative correlations with the considered outcome, indicating the need to employ a complex model that accounts for all variables and their possible synergistic interactions in risk prediction.

### 3.2. Prediction of Mortality by PCLR

We performed PCLR as first statistical approach for death prediction risk. PCA was computed with all the 19 gene variables and the 13 clinical features and the first 14 PCs have been retained (eigenvalue > 1.0) explaining approximately more than 60% of the total variance. By considering death as dependent variable and the 14 PCs as independent variables in a logistic regression model, we found significant positive association with death risk in PC1, PC3, and PC14, and negative association in PC5 and PC9 (Supplementary Table S3). Supplementary Table S4 summarizes the loadings of each PC pointing out the eigenvector values exceeding 0.3. In detail, the loadings that greatly contributed to the PCs were: PC1 (age, dementia, hypertension, ischemic stroke, heart failure, arteriopathy, *CRP rs2808635*, *CRP rs876538*); PC3 (*CRP rs2808635*, *CRP rs876538*, ventilation); PC5 (*ACE2 rs2285666*, hepatopathy, *HLA-A rs2499*, ventilation); PC9 (*CFH rs1061170*, *UCP1 rs1800592*, *APOE rs7412*, CF-carrier, diabetes); PC14 (COPD, *IL6 rs2228145*, ventilation). Despite the logistic regression model achieved a global percentage of correctness of 82.8%, the low event-per-variable ratio (EPV ~2.9), determined considering the number of outcome events (92 deaths) relative to the number of predictors (32 variables), suggests that classical multivariate regression may be constrained, while tree-based ML methods are more suitable.

### 3.3. Prediction of Hospital Length Stay by PCLR

The same approach was applied for hospital stay prediction among survivals. PCA yielded 13 PCs with an eigenvalue > 1.0 explaining approximately more than 58% of the total variance. By considering the cut-off of 15 days (median value) of hospital stay as dependent variable in a logistic regression model, we found significant positive association in PC4, PC8 and negative association in PC3 and PC6 (Supplementary Table S5). Supplementary Table S6 summarizes the loadings of each PC. The regression model achieved a global percentage of correctness of 66.5% indicating that the model has a limited predictive capability, requiring the employment of alternative approaches.

### 3.4. Prediction of Mortality by ML Models: Development and Optimization

Table 3 reports the recognized benchmarks for model screening (accuracy and AUROC) and the more ideal metrics (f1, f2, PR-AUC) for the three ML models (GBM, XGB, RF) with a 10-fold cross-validation approach.

Each metric represents the highest average score obtained when that specific metric was the optimization target during the grid search phase. Although RF achieved the highest overall accuracy (0.85), the model discrimination performance was moderate (AUROC = 0.61) specifically in the less represented class (death), accounting for high false negative count (N = 68). Under comparable PR-AUC values, XGB AUROC indicates its better predictive power under class imbalance. XGB still achieved a competitive accuracy score demonstrating its ability to generalize well across folds. For these reasons, it was selected for model construction. In particular, we were interested in improving the precision and recall values, both accounted for f1, f2, and PR-AUC calculation, to decrease false negative and false positive predictions. Comparing the f1, f2, and PR-AUC in the confusion matrix, the f1 optimization showed the lowest number of false positives (N = 26) and false negatives (N = 63) as shown in Table 4, as well as being the metric with the highest value for this model (f1 = 0.65 versus f2 = 0.60 and PR-AUC = 0.62).

Moreover, f1 optimization was more useful for the healthcare system because monitoring more patients (N_f1_ = 55 versus N_f2_ = 50 and N_PR-AUC_ = 52) may potentially save most of them, instead of losing a high number of patients due to misdiagnosis.

### 3.5. Single Analyses for XGB f1 Optimization

**Figure 2:**
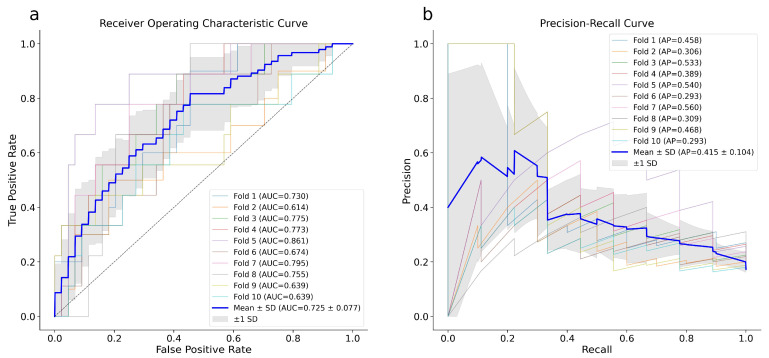
ROC (**a**) and PR (**b**) curves of the XGB model. The plotted blue curves represent the average of 10-fold cross-validation executed utilizing the whole dataset. ROC, Receiver Operating Characteristic; PR, Precision Recall.

Figure 2 shows the ROC curve (a) and the PR curve (b) for XGB f1 optimization. The model achieved a mean AUROC of 0.73 ± 0.08 and a mean PR-AUC of 0.42 ± 0.10, confirming the goodness of fit.

**Figure 3:**
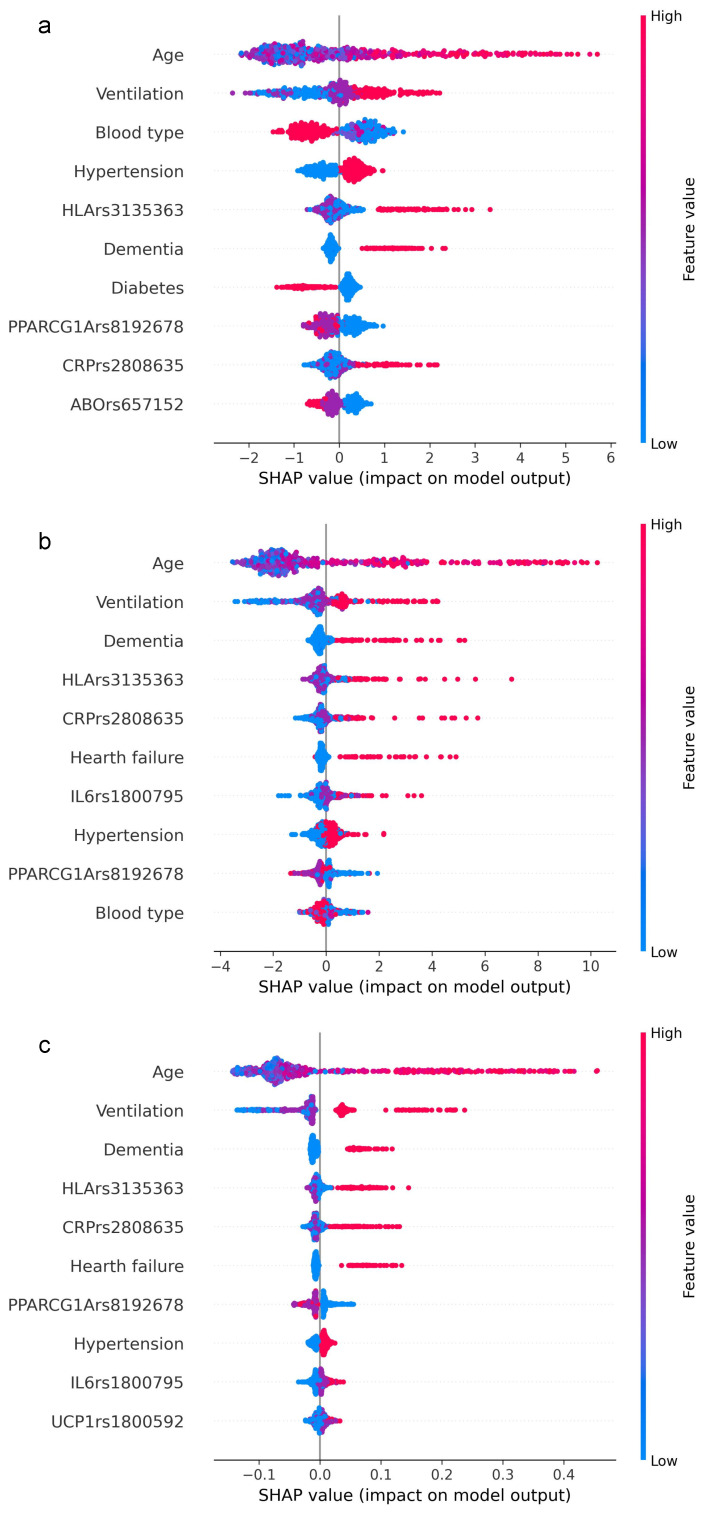
Feature importance ranking based on SHAP values. The figure shows the relative impact of the topmost features contributing to the classification in descending order for XGB (**a**), GBM (**b**), and RF (**c**). Each row (y-axis) represents a different feature, and the horizontal x-axis represents the direction of the relationship between a variable and the outcome coded as SHAP value. As demonstrated by the color bar on the right, higher values are shown in red, while lower values are shown in blue.

We delved into the feature importance results and compared the first ten important variables, with equal f1 optimization, for XGB (Figure 3a), GBM (Figure 3b), and RF (Figure 3c) to understand how and which features (genetics and clinical) contribute most to the mortality prediction.

From the comparison of the first ten variables, out of 32 computed by the models, it is evident that age and ventilation achieve the greatest values. This means that older age is consistently associated with positive SHAP values and indicates that age is the main contributor to the model decision. For all three models, age was immediately followed by the level of ventilation support at patient admission, ranging from supplemental oxygen (nasal cannula) to invasive ventilation. Although in a different order, approximately the same remaining features emerge, almost equally distributed between clinical and genetic. Interestingly, three gene variants (*HLA-DRA* rs3135363, *PPARGC1A* rs192678, *CRP* rs2808635) were found in all three algorithms. As expected, in the XGB prediction, age had the strongest predictive power, followed by ventilation, blood type, hypertension, *HLA-DPB1* rs9277356, dementia, diabetes, *PPARGC1A* rs192678, *CRP* rs2808635, and *ABO* rs657152.

Finally, to determine the weight of the mortality risk ascribable to those gene variants included among the top ten, crude ORs have been assessed under dominant and recessive inheritance models and allele frequencies for the minor allele (Table 5).

*HLA-DRA* rs3135363 yields a two-fold risk of death by the recessive model ascribing to the GG homozygous genotype significant values in the whole population (OR = 2.00; 95%CI, 1.12–3.57; *p*-value = 0.01) and in the females the risk was even higher (♀ OR = 2.80; 95%CI, 1.22–6.41; *p*-value = 0.015). The significant OR referred to *IL6* rs1800795 (OR = 1.40; 95%CI, 1.01–1.94; *p*-value = 0.04) assigns to the C allele the risk condition, slightly higher in males (♂ OR = 1.54; 95%CI, 1.00–2.38; *p*-value = 0.05).

### 3.6. Prediction of Hospital Length Stay by ML Models: Development and Optimization

The same workflow was applied to predict hospitalization duration among survivors in our dataset, another informative outcome of COVID-19 severity. Using GBM and RF algorithms, we did not find any significant relationships between the dependent and independent variables (R^2^ = 0.135 and 0.132, respectively). The complete metrics are shown in Table 6.

**Figure 4:**
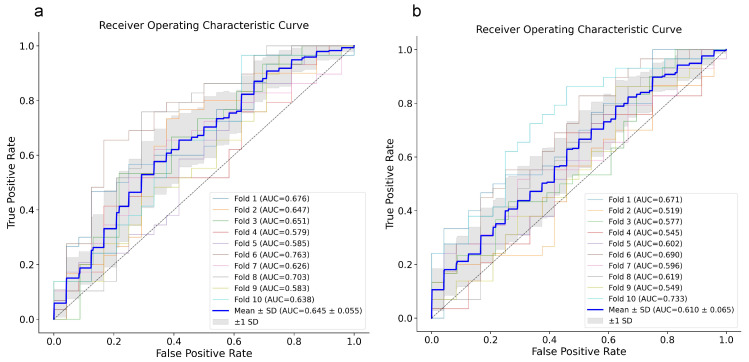
ROC curves for RF (**a**) and XGB (**b**) models. ROC curves for each 10-fold cross-validation are reported with the relative AUC and their mean ± SD (blue line). ROC, Receiver Operating Characteristic; XGB, eXtreme Gradient Boosting; RF, Random Forest.

By combining the two outcomes, assigning (1) to dead patients or patients who had longer than 15 days of hospitalization, RF and XGB models reached an AUC of around 60–65%, which means prediction slightly more than chance, but still not enough to be considered reliable predictive models (Figure 4a,b). This is also an attempt to boost the severity parameter for disease progression, which aids in rebalancing the dataset too.

## 4. Discussion

The use of big data in healthcare information systems has experienced rapid growth, with customized analyses of individual patient information to identify critical health trends and enable timely preventive care [70]. AI, including ML and big data analytics, has become a promising tool in early diagnosis and prognosis prediction by analyzing diverse datasets to manage complex diseases at single or population levels. The emergence of ever-evolving infectious diseases, such as the recent COVID-19 pandemic, underscores the pressing need for innovative tools to curb their dramatic health and economic impact [71].

The choice to use the three selected ML models was intentional and based on the characteristics of our dataset and the clinical goal of the study. Our aim was not to test all available algorithms, but to select reliable models suitable for this specific scenario. GBM, XGB and RF are well-established tree-based methods that are widely used in clinical prediction [72]. They perform well with a limited sample size, heterogeneous variables and class imbalance, as in our cohort. In addition, these models provide a good balance between predictive performance and interpretability, which is important in a clinical context where understanding the role of each feature is required. Complex models, such as deep learning approaches, usually need much larger datasets and may reduce model transparency without clear performance advantages in this setting [73]. Importantly, the main objective of this study was not algorithm benchmarking, but to assess the added value of combining genetic and clinical data within robust and clinically applicable machine learning models.

The main result of our investigation is that all the displayed mortality models achieve high values, in particular the XGB showed a better predictive power under class imbalance and a high discrimination performance specifically in the less represented class (e.g., death). XGB f1 optimization is not only the best option from a technical point of view, but also the optimization that best serves the primary purpose of the healthcare system. Specifically, the f1 metric focuses on patient quality of care, accounting the lowest number of false negative and false positive, monitoring a wide number of patients, instead of monitoring a limited number of patients that could be misdiagnosed, only addressing the economical side.

Feature importance was further analyzed for XGB, GMB and RF with f1 optimization to verify the contribution of each feature to model prediction. From the comparison of the first ten important variables, it is evident that most overlapped in all three models, and that the genetic component integrates with and supports the model predictive power, along with the clinical/epidemiological component. It should be noted that the features are selected according to the standard error, a measure of how much they improve or worsen the algorithm classification when considered, even if this does not directly correlate with the risk of death. Even though, from a clinical perspective, the presence of comorbidities, need for ventilation and older age have a greater impact on mortality risk, the genetic component does not confound the prediction; rather, it could aid in identifying peculiar cases within the vast clinical heterogeneity, enabling a more personalized approach. Since it is a complex model applied to a complex and multifactorial pathology, the feature importance results also reflect the interactions among variables. Accordingly, the ML model allowed us to screen all variables to identify the most important ones, which would require further individual analysis for a more specific assessment of their involvement in disease progression.

Regarding the top ten variables identified by the feature importance ranking of our selected model, we discuss below their implication and roles in COVID-19. As expected, aging is a major driver of mortality. In our cohort, the mean age of dead patients was about 13 years higher than survivors (80.35 y versus 67.70 y), confirming the highest correlation coefficient (0.31). Scientific evidence suggests that people older than 65 years have a greater risk of developing severe forms of COVID-19 [74]. This is mainly attributed to a weaker immune system and the presence of past or concomitant age-related diseases, leading to persistent low-grade innate immune activation [75]. The need for ventilation and the required ventilator support at hospital admission resulted in the second driver in our model decision, representing a significant risk factor for COVID-19 mortality, reflecting the severity score of respiratory compromise [76]. Patients requiring immediate mechanical ventilation, both invasive and non-invasive, are typically those with impaired gas exchange and advanced lung injury, the leading cause of death from COVID-19 [77]. Hypertension has also been widely reported as a significant risk factor for severe COVID-19 outcomes [78]. Pathophysiologically, chronic hypertension is characterized by endothelial dysfunction and vascular inflammation, conditions that can be exacerbated by SARS-CoV-2 infection, leading to multi-organ injury [79]. Furthermore, hypertensive patients present dysregulation of the renin–angiotensin–aldosterone system (RAAS), and SARS-CoV-2-mediated downregulation of ACE2 receptor may further enhance the RAAS imbalance, worsening patient conditions [80]. On the other hand, diabetes is characterized by a pathological increase in ACE2 expression, which may favor virus entry and trigger comorbidities associated with severe COVID-19 outcomes [81]. Elevated glucose levels also affect SARS-CoV-2 replication, innate and adaptive immunity, and favor hypercoagulability conditions [81]. Virus entrance is additionally influenced by blood type through the interaction of the virus with the ACE2 receptor [82,83]. In detail, blood type O appears protective against disease progression compared with non-O types [84]. Interestingly, the ABO(H)-like antigens, carried by SARS-CoV-2 envelope, may stimulate the immune response of natural ABO antibodies. This may give to type O-carriers an advantage due to the presence of both anti-A and anti-B antibodies [85,86], able to bind the Spike protein and block ACE2 entry. Interestingly, a recent report on the mechanisms by which the *CFTR* gene may influence SARS-CoV-2 infection and COVID-19 disease severity found no association between FC-carriers and more severe clinical outcomes [87]. Authors found an unexpected trend toward higher mortality among non-carrier controls compared with silent carriers stating that further studies are required. Accordingly, we detected FC-carriers exclusively among survivors (3.7%) and this subgroup, though significantly older, did not have more severe lung parameters when compared to non-carriers speculating a protective action.

As regards dementia, the literature is quite controversial on the association of dementia with enhanced risk of severe COVID-19 for several reasons. Being an age-related condition, affected patients are older, and the chance of suffering from other comorbidities is higher [88], but dementia itself remains an independent predictor of poor prognosis.

Basically, an effective immune response against viral infections is crucial for host defense; hence, HLA class II molecules present antigenic peptides to activate CD4+ T helper cells, driving cell-mediated immunity [62]. Concerning genetic data, HLA class II gene variants (e.g., rs3135363; rs9277356) have been previously shown to impact the immune response and susceptibility to disease severity by modulating the inflammation process [60,63,89,90]. Specifically, the rs3135363 variant has been linked to a more robust antibody response to hepatitis B vaccination [62], suggesting that it may influence susceptibility to infectious diseases by affecting antigen presentation and immune regulation within the MHC region. We proved that the GG genotype at rs3135363 was associated with death, with a significant 2-fold increased risk in the whole group and an almost 3-fold in the female subgroup. Our group already demonstrated different vaccine induced IgG levels in serum of patients according to *HLA-A* gene variants [38]. Severe forms of COVID-19 are mainly precipitated or triggered by an uncontrolled inflammatory response that may lead to cytokine storm [91]. C-reactive protein also plays a key role in inflammation by acting as an acute-phase reactant [92]. Elevated CRP levels are associated with a wide range of pathological conditions, including bacterial and viral infections. Its levels rise in response to inflammatory cytokines [93] and circulating levels are associated with *CRP* rs2808635. These in turn correlate with anti-SARS-CoV-2 IgG levels in COVID-19 patients, suggesting the persistence of an unresolved inflammation status [38,94]. Additionally, *PPARGC1A* rs8192678 results in Gly482Ser amino acid substitution in the PPARG coactivator-1α, a key protein of metabolic pathways. In particular, the T allele (Ser482) is associated with reduced expression and activity of the protein, leading to impaired co-activation of PPARs (Peroxisome Proliferator Activated Receptors) [95]. Given that PPARs play an anti-inflammatory role, and that SARS-CoV-2 infection downregulates PPARG coactivator-1α, the anti-inflammatory capacity is further reduced in the variant carriers. This may contribute to an unrestrained, potentially dangerous inflammatory response during COVID-19 [59]. Finally, *ABO* rs657152, which emerged from the feature importance, has been extensively studied for its association with COVID-19 severity. GWAS have identified rs657152 as a genetic risk factor for severe COVID-19, with the A allele linked to an increased risk of respiratory failure and mortality in some populations [96]. Functionally, the SNP has been linked to changes in the plasma levels of multiple proteins, including those involved in coagulation and immune response pathways. This is crucial because COVID-19 lethality is mainly due to hypercoagulability, thrombotic complications and excessive inflammation [97,98].

Although the limitations of this study include a small sample size, we previously evaluated data adequacy using multiple complementary criteria (e.g., EPV ratio and the calibration analysis shown in Section 3). The model robustness was further supported by the stability of performance across 10-fold cross validation, approach chosen instead of dividing the dataset into training and test set. Indeed, the need for external validation of the model is mandatory to confirm its performance in independent cohorts. This should address an infectious disease by using a multilayer approach and applying ML strategies to other unresolved or future complex diseases. The decision to compare different algorithms and optimization methods stems from the need to have several efficient models to recover in other scenarios of infectious diseases. For example, the Marburg virus disease (MVD), the recent, widespread human respiratory syncytial virus (RSV) [71,99], or the complex health emergency (‘Disease-X’) spread in December 2024 in a remote region of the Democratic Republic of Congo [100].

## 5. Conclusions

To build preparedness against pandemics and support the global response to widespread infections, it is important to implement intelligence-based models that incorporate genetic predispositions to predict which patient may be at higher risk of disease progression [101]. Timely and accurate identification of poor outcomes can guide physicians in prioritizing patients, allocating limited medical and economic resources more effectively, and selecting appropriate treatments. ML algorithms have revolutionized the field of healthcare by providing unprecedented opportunities to develop highly accurate models for risk analysis. This enables the precise identification of druggable molecular pathways and enhances predictive capabilities to address the myriad challenges modern healthcare systems face. These not only facilitate early diagnosis and personalized treatment plans but also optimize resource allocation and improve patient outcomes [102], reducing uncertainty and ambiguity. One practical example is the respiratory failure, recommended for assisted ventilation, a scarce resource during the pandemic phase [103]. We can speculate that our model could prioritize the resource allocation to patients not only via clinical criteria alone. Finally, a critical challenge in predictive modeling is selecting the model that best balances data fit and clinical relevance. This highlights the importance of choosing the perfect algorithm and the best optimization avoiding false negative bad outcome predictions.

## Figures and Tables

**Table 3:** Metric values for GBM, XGB and RF.

	Accuracy	AUROC	f1	f2	PR-AUC
GBM	0.83 (±0.03)	0.61 (±0.07)	0.63 (±0.09)	**0.61 (±0.08)**	0.62 (±0.09)
XGB	0.83 (±0.03)	**0.63 (±0.04)**	**0.65 (±0.05)**	0.60 (±0.05)	0.62 (±0.07)
RF	**0.85 (±0.04)**	0.61 (±0.06)	0.63 (±0.09)	**0.61 (±0.07)**	**0.68 (±0.12)**

GBM, Gradient Boosting Machine; XGB, eXtreme Gradient Boosting; RF, Random Forest; PR-AUC, Precision Recall-Area Under the Curve. The highest values are reported in bold.

**Table 4:** Confusion matrix of f1, f2, and PR-AUC metrics for XGB.

**f1**	**Predicted Negative (0)**	**Predicted Positive (1)**	**Error**
actually negative (0)	414	26	0.0591
actually positive (1)	63	29	0.3152
total	477	55	0.1673
**f2**	**predicted negative (0)**	**predicted positive (1)**	**error**
actually negative (0)	413	27	0.0614
actually positive (1)	69	23	0.2500
total	482	50	0.1805
**PR-AUC**	**predicted negative (0)**	**predicted positive (1)**	**error**
actually negative (0)	411	29	0.0659
actually positive (1)	69	23	0.2500
total	480	52	0.1842

**Table 5:** OR results for the gene variants in the whole population.

OR (95%CI); *p*-Value	*HLA-DRA* rs3135363 (G)	*IL6* rs1800795 (C)	*CRP* *rs2808635* (G)	*PPARGC1A* *rs7192678 (T)*	*ABO* *rs657152 (A)*	*UCP1* *rs1800592 (C)*
Dominant model	1.00 (0.64–1.58) n.s.	1.53 (0.97–2.42) 0.07	1.12 (0.72–1.75) n.s.	0.75 (0.48–1.18) n.s.	1.21 (0.76–1.94) n.s.	0.97 (0.62–1.52) n.s.
Recessive model	2.00 (1.12–3.57) **0.01**	1.60 (0.82–3.11) n.s.	1.75 (0.93–3.30) 0.08	1.14 (0.58–2.24) n.s.	1.06 (0.56–2.03) n.s.	1.38 (0.64–3.00) n.s.
Allele frequency	1.03 (0.73–1.44) n.s.	1.40 (1.01–1.94) **0.04**	1.24 (0.88–1.74) n.s.	0.88 (0.63–1.24) n.s.	1.12 (0.81–1.55) n.s.	1.04 (0.73–1.48) n.s.

OR, Odds Ratio; CI, Confidence Interval; n.s., not significant; *p*-values < 0.05 are reported in bold. In brackets: the minor allele.

**Table 6:** GBM and RF model metrics for days of hospitalization.

	MSE	RMSE	MAE	MAPE	R^2^
**GBM**	321.22	17.31	11.74	0.97	0.135
**RF**	318.59	17.27	11.67	0.96	0.132

GBM, Gradient Boosting Machine; RF, Random Forest; MSE, Mean Squared Error; RMSE, Root Mean Squared Error; MAE, Mean Average Error; MAPE, Mean Absolute Percentage Error; R2, coefficient of determination.

## Data Availability

The data supporting this study’s findings are available from the corresponding authors upon reasonable request, pending internal agreement among the participating centers.

## Supplementary Materials

The following supporting information can be downloaded at: https://www.mdpi.com/article/10.3390/diagnostics16040583/s1, Table S1: Hyperparameter values explored during grid search; Table S2: Optimal hyperparameter configurations identified through grid search for GBM, XGB and RF under different optimization metrics; Table S3: PCLR analysis; Table S4: Loadings of PCs; Supplementary Table S5: PCLR analysis; Table S6: Loadings of PCs.

## Abbreviations

The following abbreviations are used in this manuscript:

ABO	Alpha 1-3-N-acetylgalactosaminyltransferase and alpha 1-3-galactosyltransferase
ACE2	Angiotensin converting enzyme 2
AI	Artificial Intelligence
APOE	Apolipoprotein E
AUC	Area Under the Curve
CCR2	C-C motif Chemokine Receptor 2
CFH	Complement Factor H
CFTR	Cystic Fibrosis Transmembrane Conductance Regulator
CI	Confidence Interval
COPD	Chronic Obstructive Pulmonary Disease
COVID-19	Coronavirus Disease 19
CRP	C-Reactive Protein
ECV	ExtraCorporeal Ventilation
GBM	Gradient Boosting Machine
GWAS	Genome-Wide Association Study
HGI	Host Genetics Initiative
HLA	Human Leukocyte Antigen
ICU	Intensive Care Unit
IL6	Interleukin6
IRB	Institutional Review Board
LZTFL1	Leucine zipper transcription factor-like 1
MAE	Mean Average Error
MAPE	Mean Absolute Percentage Error
ML	Machine Learning
MSE	Mean Squared Error
MVD	Marburg Virus Disease
NCBI	National Center for Biotechnology Information
NIV	Non-Invasive Ventilation
OAS3	3′,5′-Oligoadenylate Synthase 3
OR	Odds Ratio
PCA	Principal Component Analysis
PCLR	Principal Component Logistic Regression
PPARGC1A	PPARG coactivator 1 alpha
PPARs	Peroxisome Proliferator Activated Receptors
PR	Precision Recall
RAAS	Renin–Angiotensin–Aldosterone System
RF	Random Forest
RMSE	Root Mean Squared Error
ROC	Receiver Operating Characteristic
SARS-CoV-2	Severe Acute Respiratory Syndrome Coronavirus 2
SE	Standard Error
SHAP	SHapley Additive exPlanations
SNP	Single Nucleotide Polymorphism
SPSS	Statistical Package for the Social Sciences
TP53	Tumor Protein p53
UCP1	Uncoupling Protein 1
XGB	eXtreme Gradient Boosting
