# Supplementary material for: Integrating Host Genetics and Clinical Setting in Machine Learning Models: Predicting COVID-19 Prognosis for Healthcare Decision-Making (The FeMiNa Study)
Source: Diagnostics (Basel). 2026 Feb 15;16(4):583. doi: 10.3390/diagnostics16040583 (PMC12939118; doi:10.3390/diagnostics16040583)
Supplement: Supplementary file 1 [file diagnostics-16-00583-s001.zip › diagnostics-4063679-supplementary.pdf]

Supplementary Table S1. Hyperparameter values explored during grid search.

Model	Hyperparameter	Values explored
RF	max_depth	3, 7
RF	max_features	None
RF	min_samples:leaf	5, 8, 10
RF	n_estimators	50, 100, 200, 400, 1000
GBM	learning_rate	0.01, 0.05, 0.10, 0.20
GBM	max_depth	3, 7, 20
GBM	max_features	0.5, 0.8, 1.0
GBM	n_estimators	50, 100, 150, 400
GBM	subsample	0.8, 0.9, 1.0
XGB	booster	dart, gbtree
XGB	colsample_bytree	0.5, 0.7
XGB	learning_rate	0.01, 0.05, 0.10
XGB	max_depth	3, 5, 7
XGB	subsample	0.8, 1.0
XGB	n_estimators	600

RF, Random Forest; GBM, Gradient Boosting Machine; XGB, eXtreme Gradient Boosting.

Supplementary Table S2. Optimal hyperparameter configurations identified through grid search for GBM, XGB and RF under different optimization metrics.

Model	Metric	booster	colsample_ bytree	learning_ rate	max_ depth	subsample	n_ estimators	max_ features	min_ samples:leaf
GBM	f1	-	-	0.10	3	0.8	50	1.0	-
GBM	f2	-	-	0.20	3	0.8	50	0.8	-
GBM	PR-AUC	-	-	0.20	15	0.8	50	1.0	-
XGB	f1	gbtree	0.5	0.10	7	0.8	600	-	-
XGB	f2	dart	0.7	0.05	3	0.8	600	-	-
XGB	PR-AUC	dart	0.7	0.10	5	0.8	600	-	-
RF	f1	-	-	-	7	-	200	None	5
RF	f2	-	-	-	3	-	200	None	10
RF	PR-AUC	-	-	-	7	-	200	None	10

RF, Random Forest; GBM, Gradient Boosting Machine; XGB, eXtreme Gradient Boosting.

Supplementary Table S3. PCLR analysis

PC	OR (95%CI)	P-value
PC1	2.239 (1.707-2.937)	**<0.001**
PC2	1.039 (0.805-1.339)	0.771
PC3	1.583 (1.205-2.079)	**<0.001**
PC4	1.122 (0.874-1.439)	0.366
PC5	0.722 (0.546-0.954)	**0.022**
PC6	1.059 (0.808-1.386)	0.678
PC7	1.164 (0.901-1.505)	0.245

PC8	0.832 (0.649-1.066)	0.146
PC9	0.696 (0.531-0.913)	**0.009**
PC10	1.017 (0.772-1.339)	0.905
PC11	0.898 (0.696-1.157)	0.403
PC12	0.911 (0.710-1.167)	0.459
PC13	0.801 (0.615-1.044)	0.100
PC14	1.421 (1.093-1.848)	**0.009**

PC, Principal Component; OR, Odds Ratio; CI, Confidence Interval.

Supplementary Table S4. Loadings of PCs

Variables	PC1	PC2	PC3	PC4	PC5	PC6	PC7	PC8	PC9	PC10	PC11	PC12	PC13	PC14
Age	**0.718**	0.126	0.291	0.106	-0.091	0.032	0.012	-0.146	0.015	-0.036	0.034	-0.091	-0.085	0.071
Dementia	**0.535**	0.122	0.227	0.026	0.111	0.088	-0.151	-0.190	-0.161	0.126	0.166	-0.201	0.221	0.145
Hypertension	**0.522**	-0.001	0.133	0.181	-0.020	-0.238	0.171	0.116	0.057	-0.123	0.076	0.141	-0.144	0.282
Ischemic stroke	**0.459**	0.113	0.184	-0.128	0.028	0.107	-0.111	0.360	-0.133	0.052	-0.116	0.133	0.112	-0.021
Hearth failure	**0.438**	-0.003	0.134	-0.049	-0.234	0.113	-0.035	0.294	0.128	-0.186	-0.111	-0.107	0.217	-0.118
Arteriopathy	**0.334**	-0.252	-0.019	-0.297	-0.146	-0.164	0.284	0.205	0.004	-0.058	-0.067	0.105	0.114	0.138
ABOrs657152	0.147	-0.834	0.178	0.046	-0.029	0.098	-0.157	-0.033	-0.008	0.096	0.028	0.071	-0.128	-0.043
Blood type	-0.070	0.809	-0.210	-0.050	0.038	-0.137	0.222	0.006	-0.001	-0.213	-0.030	-0.067	0.111	0.058
CRPrs876538	**-0.319**	0.063	**0.754**	0.062	-0.049	-0.152	0.212	0.104	0.031	0.213	-0.017	0.061	-0.010	-0.124
CRPrs2808635	**-0.340**	0.180	**0.712**	0.102	-0.070	-0.166	0.194	0.135	-0.067	0.168	0.094	0.060	0.001	-0.016
CCR2rs34041956	-0.028	-0.009	0.067	0.570	0.002	0.451	0.237	0.041	-0.107	-0.036	-0.216	0.064	-0.030	0.081
LZTFL1rs35044562	-0.115	0.016	-0.061	0.467	0.174	0.402	0.176	0.215	-0.132	-0.048	-0.340	0.096	0.065	0.130
OAS3rs10735079	-0.030	-0.198	-0.169	0.428	0.148	-0.213	-0.025	-0.020	0.096	-0.237	0.051	-0.206	0.076	-0.078
TP53rs1042522	-0.088	-0.058	0.105	-0.340	0.252	0.036	-0.283	0.262	-0.092	-0.316	-0.227	0.211	0.134	0.138
ACE2rs2285666	0.041	-0.091	-0.154	0.106	**0.539**	-0.140	0.060	0.130	-0.151	0.216	0.121	-0.151	0.321	-0.094
Hepatopathy	0.155	-0.073	-0.093	-0.079	**0.481**	-0.037	0.059	-0.204	-0.164	0.305	-0.176	0.392	0.024	-0.133
HLAArs2499	-0.113	0.023	-0.043	-0.009	**-0.446**	0.141	-0.159	-0.216	0.198	0.205	-0.060	0.099	0.387	-0.186
APOErs429358	-0.109	-0.044	-0.078	-0.253	0.058	0.471	0.165	0.149	0.052	0.018	0.307	-0.067	-0.145	-0.027
HLArs9277356	0.028	0.025	0.026	-0.397	0.296	0.269	0.479	0.030	0.127	0.046	0.229	0.009	0.014	0.044
HLArs3135363	0.058	0.255	0.000	0.017	0.102	-0.139	-0.372	0.119	0.220	0.336	-0.320	-0.008	-0.129	0.038
CFHrs1061170	0.020	0.059	-0.156	-0.096	-0.267	0.104	-0.030	0.203	**-0.452**	0.283	-0.057	-0.083	0.304	-0.019
UCP1rs1800592	-0.083	0.132	0.203	0.063	0.060	-0.155	-0.239	-0.180	**-0.431**	-0.303	0.129	0.212	-0.004	0.126
APOErs7412	-0.138	-0.033	0.151	-0.090	-0.028	0.266	-0.041	-0.069	**0.413**	-0.280	-0.145	0.159	0.118	0.135
CFcarrier	-0.065	0.002	0.132	0.118	0.288	-0.086	-0.229	-0.013	**0.377**	0.101	-0.059	-0.233	0.158	0.275
PPARCG1Ars8192678	0.051	0.174	-0.240	0.181	-0.135	0.089	-0.094	0.336	0.269	0.378	0.240	0.133	0.002	0.233
COPD	0.263	-0.027	-0.121	-0.011	-0.142	-0.165	0.389	-0.120	-0.021	0.016	-0.434	-0.223	-0.098	**-0.324**
Neoplasm	0.211	0.255	-0.066	-0.002	-0.056	0.142	0.004	-0.459	0.084	0.094	-0.029	0.501	0.012	-0.026
Diabetes	0.220	-0.099	-0.128	0.269	0.100	-0.288	0.264	0.051	**0.306**	-0.025	0.214	0.307	0.229	-0.137
IL6rs1800795	-0.063	-0.133	0.174	0.045	-0.046	0.166	0.123	-0.393	-0.067	-0.038	0.103	-0.169	0.460	0.220
Sex	0.289	0.257	0.091	0.084	0.133	0.331	-0.159	-0.119	-0.024	0.075	0.151	-0.183	-0.347	-0.140
IL6rs2228145	0.032	0.101	-0.043	0.272	-0.080	0.073	-0.223	0.250	-0.073	-0.214	0.376	0.196	0.088	**-0.445**
Ventilation	-0.103	-0.121	**-0.316**	0.111	**-0.333**	-0.167	0.107	0.013	-0.238	0.145	0.110	0.098	-0.143	**0.442**

The PCs identified through PCLR analysis are highlighted in light grey. In bold the loadings exceeding the cut-off value > 0.30. PC, Principal Component; CF, Cystic fibrosis; COPD, Chronic Obstructive Pulmonary Disease.

Supplementary Table S5. PCLR analysis

PC	OR (95%CI)	P-value
PC1	1.125 (0.904-1.400)	0.290
PC2	0.835 (0.670-1.042)	0.111
PC3	0.788 (0.632-0.983)	**0.035**
PC4	1.413 (1.130-1.765)	**0.002**
PC5	0.829 (0.667-1.030)	0.091
PC6	0.763 (0.612-0.951)	**0.016**
PC7	1.187 (0.951-1.483)	0.130
PC8	1.295 (1.037-1.617)	**0.022**
PC9	0.924 (0.743-1.150)	0.479
PC10	1.049 (0.843-1.305)	0.670
PC11	1.009 (0.809-1.258)	0.939
PC12	1.016 (0.818-1.262)	0.887
PC13	1.137 (0.915-1.414)	0.248

PC, Principal Component; OR, Odds Ratio; CI, Confidence Interval.

Supplementary Table S6. Loadings of PCs

Variables	PC1	PC2	PC3	PC4	PC5	PC6	PC7	PC8	PC9	PC10	PC11	PC12	PC13
Age	0.646	0.360	0.167	0.114	0.108	-0.114	0.051	-0.053	-0.010	0.005	-0.051	-0.082	-0.072
Hypertension	0.521	0.100	0.136	0.208	-0.121	-0.217	-0.070	0.223	-0.075	-0.197	0.036	-0.057	0.049
Dementia	0.449	0.312	0.122	0.018	0.153	0.054	-0.008	-0.053	0.064	0.374	0.206	0.096	-0.127
Arteriopathy	0.422	-0.202	0.090	-0.266	-0.170	-0.113	0.175	0.179	-0.189	-0.146	0.127	0.061	-0.007
Hearth failure	0.407	-0.005	0.190	-0.008	0.248	-0.034	0.039	0.158	-0.204	-0.215	0.287	0.044	0.044
Ischemic stroke	0.393	0.225	0.145	-0.167	0.139	0.132	-0.104	**0.350**	0.117	0.059	-0.016	0.165	0.024
Diabetes	0.356	-0.110	-0.071	0.254	-0.339	-0.137	0.046	-0.176	-0.047	-0.049	0.243	0.106	0.251
ABOrs657152	0.154	-0.575	**0.619**	0.005	0.179	-0.021	0.056	-0.055	0.138	0.042	-0.130	-0.083	0.091
Blood type	-0.033	0.560	**-0.597**	-0.036	-0.263	0.024	-0.033	0.030	-0.256	-0.069	0.117	0.096	-0.048
CRPrs876538	-0.431	0.404	**0.527**	0.231	-0.192	-0.215	0.148	0.105	0.133	-0.059	0.031	0.027	0.045
CRPrs2808635	-0.430	0.448	**0.455**	0.245	-0.183	-0.202	0.137	0.221	0.026	0.007	0.080	-0.027	-0.015
Ventilation	-0.162	-0.292	**-0.300**	0.136	0.020	-0.265	0.099	0.289	-0.012	0.042	0.035	0.016	0.028
CCR2rs34041956	0.017	-0.006	0.000	**0.580**	0.123	**0.395**	0.321	0.085	0.013	-0.115	-0.044	0.164	-0.095
OAS3rs10735079	0.120	-0.350	-0.162	**0.450**	-0.047	0.087	-0.159	-0.165	-0.085	-0.058	0.084	-0.132	-0.281
HLArs9277356	0.018	0.118	0.066	**-0.359**	-0.332	**0.339**	0.339	0.028	0.110	-0.059	0.209	-0.211	0.056
APOErs429358	-0.069	-0.049	-0.034	**-0.357**	0.071	**0.326**	0.313	0.192	0.125	-0.097	0.078	-0.266	0.058
Hepatopathy	0.236	0.027	0.084	-0.055	-0.389	0.234	-0.173	-0.004	0.259	0.269	-0.241	0.173	0.310
ACE2rs2285666	0.037	-0.176	-0.026	0.132	-0.366	0.273	-0.279	0.216	0.153	0.341	0.226	-0.005	0.154
COPD	0.336	-0.114	-0.057	0.043	-0.344	-0.195	0.256	-0.092	-0.062	-0.089	-0.264	0.108	-0.015
LZTFL1rs35044562	-0.043	-0.102	-0.093	**0.441**	0.080	**0.487**	0.096	0.236	0.102	-0.147	-0.018	0.202	0.135
TP53rs1042522	-0.094	0.020	0.205	-0.270	0.153	0.232	-0.343	0.060	-0.328	-0.067	0.044	0.308	0.030
CFHrs1061170	-0.045	-0.036	-0.147	-0.025	0.293	-0.114	0.168	**0.427**	0.032	0.299	0.061	0.351	-0.218
Neoplasm	0.209	0.313	-0.158	0.086	0.119	0.080	0.274	**-0.364**	0.148	0.129	-0.198	0.106	0.157

PPARCG1Ars8192678	0.075	-0.007	**-0.325**	-0.003	0.199	-0.193	0.027	0.149	0.472	-0.018	0.326	-0.198	0.142
HLArs3135363	0.062	0.172	-0.081	-0.055	0.191	-0.090	-0.363	-0.041	0.418	-0.302	-0.092	0.194	0.026
UCP1rs1800592	-0.076	0.191	0.067	0.114	0.159	0.030	-0.295	0.056	-0.309	0.301	-0.204	-0.233	0.187
IL6rs1800795	-0.026	-0.035	0.183	-0.028	0.023	0.149	0.238	**-0.334**	-0.182	0.394	0.323	0.096	-0.234
APOErs7412	-0.162	0.097	0.128	-0.074	0.156	0.224	0.026	-0.262	-0.157	-0.389	0.164	0.248	0.285
CFcarrier	-0.025	0.082	0.147	0.138	-0.002	0.064	-0.367	-0.253	0.253	-0.104	0.440	-0.121	-0.170
Sex	0.219	0.294	-0.049	0.055	0.243	0.273	0.054	-0.005	0.057	-0.064	-0.243	-0.415	-0.159
IL6rs2228145	0.017	0.040	-0.067	0.216	0.200	0.006	-0.026	0.132	-0.370	0.097	0.134	-0.329	0.471
HLAArs2499	-0.102	-0.012	-0.122	0.004	0.363	-0.265	0.241	-0.273	0.122	0.175	0.121	0.141	0.374

The PCs identified through PCLR analysis are highlighted in light grey. In bold the loadings exceeding the cut-off value > 0.30. PC, Principal Component; CF, Cystic fibrosis; COPD, Chronic Obstructive Pulmonary Disease.
